# CSF1R as a Therapeutic Target in Bone Diseases: Obvious but Not so Simple

**DOI:** 10.1007/s11914-022-00757-4

**Published:** 2022-10-05

**Authors:** David A. Hume, Lena Batoon, Anuj Sehgal, Sahar Keshvari, Katharine M. Irvine

**Affiliations:** Mater Research Institute-University of Queensland, Translational Research Institute, 37 Kent Street, Woolloongabba, QLD 4102 Australia

**Keywords:** Osteoclast, Macrophage, Osteoporosis, CSF1R, Homeostasis

## Abstract

**Purpose of Review:**

The purpose of the review is to summarize the expression and function of CSF1R and its ligands in bone homeostasis and constraints on therapeutic targeting of this axis.

**Recent Findings:**

Bone development and homeostasis depends upon interactions between mesenchymal cells and cells of the mononuclear phagocyte lineage (MPS), macrophages, and osteoclasts (OCL). The homeostatic interaction is mediated in part by the systemic and local production of growth factors, macrophage colony-stimulating factor (CSF1), and interleukin 34 (IL34) that interact with a receptor (CSF1R) expressed exclusively by MPS cells and their progenitors. Loss-of-function mutations in CSF1 or CSF1R lead to loss of OCL and macrophages and dysregulation of postnatal bone development. MPS cells continuously degrade CSF1R ligands via receptor-mediated endocytosis. As a consequence, any local or systemic increase or decrease in macrophage or OCL abundance is rapidly reversible.

**Summary:**

In principle, both CSF1R agonists and antagonists have potential in bone regenerative medicine but their evaluation in disease models and therapeutic application needs to carefully consider the intrinsic feedback control of MPS biology.

## Introduction

The essential requirement for macrophage colony-stimulating factor (CSF1) in bone development became evident with the identification of causal loss-of-function mutations in the *Csf1* gene in mice and rats [1–3] that were associated with severe osteopetrosis. There are also isolated reports of CSF1 deficiency in human malignant osteopetrosis [4] although this is more commonly associated with mutations in genes expressed specifically in osteoclasts (OCL) and required for the process of bone resorption [5]. The bone developmental defect in CSF1 deficiency in mice is associated with the loss of bone-resorbing OCL [6]. Conversely, genetic studies of Paget’s disease, a disorder of excessive OCL function, revealed an association with the human *CSF1* locus [7]. In mice, the osteopetrosis and OCL deficiency correct with age [8, 9]. CSF1 signals via a plasma membrane tyrosine kinase receptor encoded by the *Csf1r* gene. Homozygous mutation of *Csf1r* in mice and rats is also associated with osteopetrosis [10, 11•]. The rather more severe phenotype of the receptor mutation in mice and the age-dependent correction in CSF1-deficient models likely reflect the contribution of a second ligand, interleukin 34 (IL34), which binds to an overlapping site on the receptor and can complement CSF1 deficiency when expressed as a transgene [12]. By contrast, mutation of *Csf1* in the toothless (*tl/tl*) rat is associated with unremitting osteopetrosis and OCL deficiency but a much less severe effect on postnatal somatic growth than in mice [3, 13]. The importance of species differences is discussed further below.

Osteoporosis is commonly attributed to an imbalance between the physiologically coupled processes of osteoclastic bone resorption and osteoblastic bone formation [14, 15, 16•]. Current therapies aimed at arresting bone loss, bisphosphonates and specific antibodies, are targeted against OCL [17, 18]. The appropriate balance between bone resorption and formation/calcification is clearly also crucial in acute osteolysis in chronic inflammation, infection, and malignancy [19] and conversely in bone regeneration in response to fracture. Given the phenotypic consequences of CSF1 and CSF1R mutations during development, these molecules would appear as obvious therapeutic targets for bone-related pathologies and regenerative medicine. To assess both the opportunities and the risks of such a strategy, it is necessary to have an understanding of the expression, regulation, and function of the CSF1R gene and both of its agonists in bone. This review provides an overview of current knowledge.

## CSF1R Protein Expression is Restricted to Mononuclear Phagocytes

A key question when considering CSF1R as a therapeutic target, and in the interpretation of the biological activity of agonists, antagonists, and mutations, is the localization of expression. The transcription of the *Csf1r* gene in vivo has been tracked through the generation and analysis of *Csf1r* reporter transgenes in mice, rats, and chickens [20–25], and the molecular basis of transcriptional regulation has been reviewed elsewhere [26]. *Csf1r* mRNA is expressed by the earliest phagocytes generated in the yolk sac [27] and the *Csf1r* reporter transgenes are expressed by tissue macrophages throughout the developing embryo [25, 28]. The expression of *Csf1r* mRNA tracks with expression of other macrophage markers in transcriptome analysis of tissue macrophages [29] and reflects the progressive expansion of tissue macrophages during embryo development and further increases in the postnatal period [30]. In adult mice, *Csf1r* mRNA and protein are absent from pluripotent hematopoietic stem cells in the bone marrow [31, 32•, 33] and induced during differentiation/lineage commitment in myeloid progenitors. *Csf1r* mRNA is expressed by blood monocytes, granulocytes, tissue macrophages, dendritic cells, and OCL [33–35]. In granulocytes, *Csf1r* mRNA is co-expressed with several other macrophage-specific transcripts that are not translated into protein [34].

**Fig. 1:**
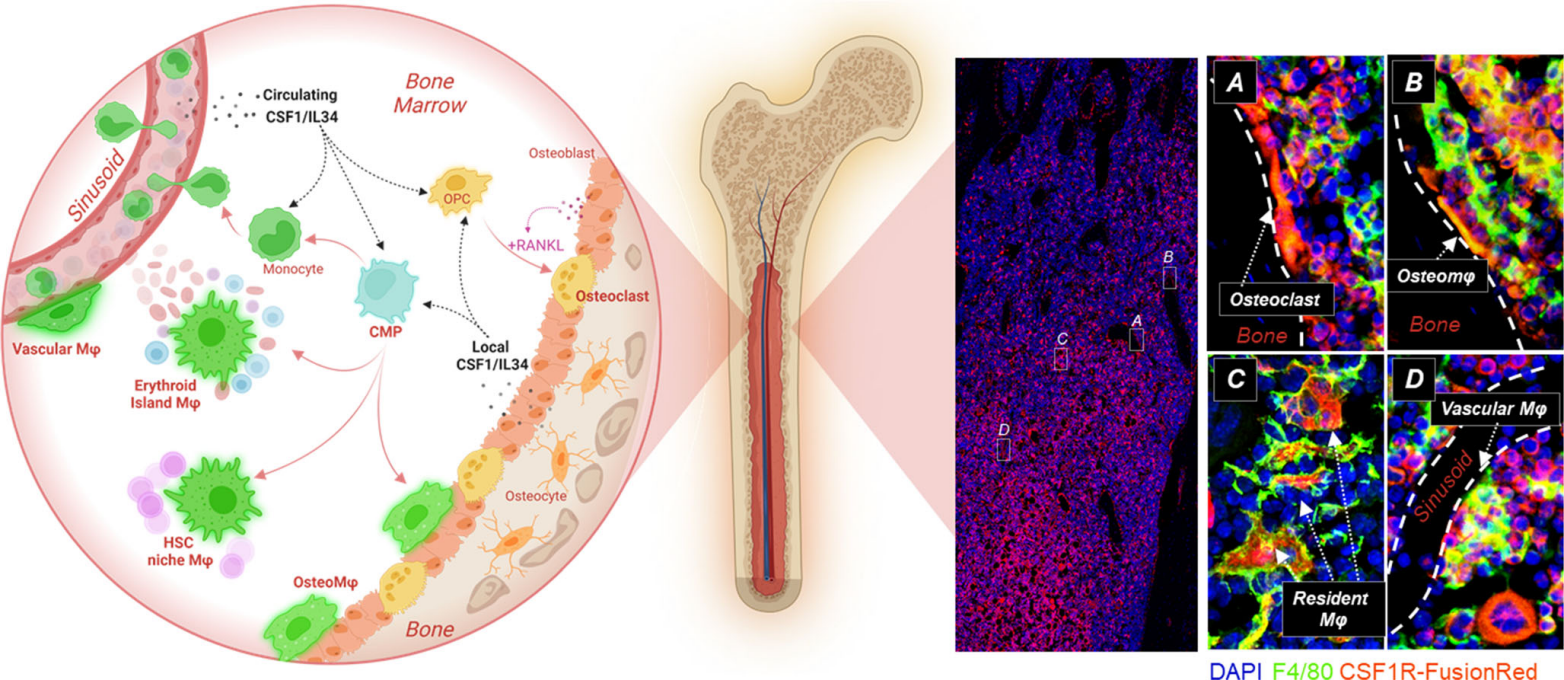
The mononuclear phagocyte populations of mouse bone. The schematic at left highlights the diversity of mononuclear phagocyte populations (osteoclasts, monocyte, macrophages (Mϕ), and committed progenitors (CMP, OMP)) found in mouse bone and the local and systemic sources of growth factors CSF1 and IL34. The images at right show the co-localization of the *Csf1r*-FusionRed transgene [32•] and the macrophage-restricted F4/80 antigen [45•]. The low power image confirms that the majority of bone marrow hematopoietic and mesenchymal (osteoblast, fibroblast, adipocyte, endothelial) cells lack expression of the FusionRed reporter [32•]. Osteoclasts (Panel **A**) express FusionRed but lack F4/80 [45•]. Mϕ populations associated with the bone surface (**B**), hematopoietic islands (**C**), and sinusoids (**D**) express both FusionRed and F4/80. F4/80 is relatively low on monocytes. Panels **A** and **D** contain multiple FusionRed-positive mononuclear cells; presumptive monocytes; and their progenitors. At bottom of Panel **D**, the large FusionRed-positive, F4/80-negative cell is a megakaryocyte

There have been multiple reports of expression of *Csf1r* mRNA or CSF1R protein outside of the myeloid lineages including developing neurons, intestinal and renal epithelial cells, and smooth muscle and mesangial cells [36, 37]. These reports are inconsistent with other evidence (reviewed in [38]). Expression outside the myeloid lineages is not supported by localization of any of the *Csf1r* reporter transgenes in any species, nor in situ localization of *Csf1r* mRNA [27, 39]. To address the issue finally, we generated a knock-in transgene that reports CSF1R protein expression [32•]. Visualization in bone marrow revealed expression in megakaryocytes, which had not previously been appreciated and may be relevant to thrombocytopenia observed in CSF1-treated animals and patients [38]. Figure 1 shows a schematic view of the MPS of bone, and images of the Csf1r-FusionRed transgene in mouse bone marrow, highlighting distinct MPS subpopulations associated with specific niches on the bone surface and within the marrow.

Outside of the marrow, expression of the CSF1R-FusionRed reporter was restricted to macrophages [32•]. There is no evidence of expression of CSF1R protein outside of the macrophage lineage in any organ at any time in development. All of the *Csf1r* reporters analyzed in multiple species highlight the abundance and regular distribution of resident macrophages in every tissue in the body, which is an important consideration when contemplating CSF1R as a therapeutic target.

In the specific case of bone, Wittrant et al. [40] claimed that *Csf1r* mRNA and protein were detectable in mouse calvarial osteoblasts and reported direct effects of CSF1 administration on osteoblast function. These workers excluded contamination of their cultures by monocyte-macrophages on the basis of flow cytometry analysis of macrophage surface markers on cells harvested by trypsinization. However, macrophage adhesion to plastic is not trypsin-sensitive and the authors did not show direct staining of their cultures. The expression of *Csf1r* mRNA in osteoblasts or any other mesenchymal population was excluded by Dai et al. [41] and it is not evident in published single-cell RNA sequencing (scRNA-seq) profiles of mouse bone marrow stromal cell populations [42, 43]. Chang et al. [44] resolved this conflict. They identified macrophages as a major persistent contaminant of standard mouse calvarial osteoblast cultures, detecting both *Csf1r* and *Adgre1* (F4/80) mRNA and F4/80 protein in situ*.* These studies led to the characterization of a resident bone macrophage population, termed osteomacs, which express CD169 (*Siglec 1*), line the surface of bone (see Fig. 1), and contribute to bone homeostasis and repair independently of OCL [44, 45•].

One exception to the macrophage-restricted expression of CSF1R occurs in the placenta. Visvader and Verma [46] first demonstrated that placental trophoblasts utilize a distinct upstream promoter to drive expression of *CSF1R* in the human placenta. This promoter in humans lies some 25kb upstream of the macrophage transcription start site (TSS), within the 3′ end of the upstream *PDGFRB* locus. An alternative non-coding exon splices into an acceptor site upstream of the first coding exon. In mice, *Csf1r* mRNA was detected in the first trophoblast precursors in the ectoplacental cone [47] and was abundant in mature placenta [27] but the precise transcriptional regulation is not conserved. In mice, the major TSS in placenta lie within 500 bp of the macrophage TSS again encoding alternative 5′ UTR exons [24]. Interestingly, this distal promoter region contains the major TSS utilized selectively by OCL and may also have essential enhancer activity for some tissue macrophage populations [48]. Our ongoing studies of *Csf1r* transcriptional regulation raised a surprising conundrum. Germ-line deletion of a highly conserved enhancer (Fms intronic regulatory element, or FIRE) in the first intron of the mouse gene (*Csf1r*^∆FIRE/∆FIRE^ ) led to the selective loss of several tissue macrophage populations and abolished expression of *Csf1r* mRNA and protein in bone marrow progenitors and blood monocytes but had no effect on OCL numbers or bone density [49]. CSF1R-deficient animals and human patients are clearly OCL-deficient and anti-CSF1R antibody also blocks OCL differentiation in osteoblast co-cultures in vitro [50]. Conditional deletion of *Csf1r* in *Tnfrsf11a* (*Rank*)–positive cells also ablates OCL development in mice [51•]. Lineage tracing studies suggested that erythro-myeloid progenitors (EMP) initially seed OCL in the embryo, and ongoing OCL maintenance is supported by fusion of HSC-derived monocytes. The latter cells may rescue OCL deficiencies associated with loss of *Csf1r* in EMP [51•].

Since resident macrophages and OCL are fragmented during disaggregation [52], the analysis of the *Csf1r* hypomorphic marrow may have excluded these populations. In common with other macrophage populations in the mutant mice, they probably utilize other enhancers to support *Csf1r* transcription [49]. In the case of OCL, there is also the unique *Csf1r* promoter. Transcription factors that might bind to the OCL-specific upstream element to promote *Csf1r* transcription have not been identified. One candidate is the master regulator NFATC1 [53]. The transcription factor PPARG is essential for osteoclastogenesis and in turn regulates *Fos*, which is also required [54]. A seminal study in this area indicated that committed OCL progenitors may be enriched for expression of *Pparg* [55]. Published Chip-Seq data on bone marrow–derived macrophages (BMDM) indicate that PPARG binds to FIRE and to multiple other distal elements in a ligand-independent manner and that binding is up-regulated by interleukin 4 [56]. Research in our group and others identified unique roles for the transcription factor MITF in osteoclastogenesis [57–59]. MITF interacts genetically and physically with the macrophage transcription factor PU.1 (*Spi1*) [58, 59]. We are currently exploring the removal of the OCL-specific promoter region from the mouse germ-line. The loss of CSF1R in bone marrow did not impact on expression of FLT3 in progenitor cells [49] and it is possible that FLT3L can compensate in OCL differentiation in mice independently of CSF1R expression. The reciprocal compensation has been described in mouse dendritic cell differentiation, where CSF1R can compensate for the lack of FLT3 [60]. Lean et al. [61] suggested that FLT3L is responsible for age-dependent recovery of OCL in CSF1-deficient mice, although this was prior to the discovery of IL34. Indeed, Nakamichi et al. [62] provided evidence for an IL34-dependent OCL progenitor population in the spleen of *op/op* mice. The effect of a compound *Csf1/Il34* mutation in mice or rats has not yet been reported.

## Expression and Function of CSF1 and IL34 in Bone

**Fig. 2:**
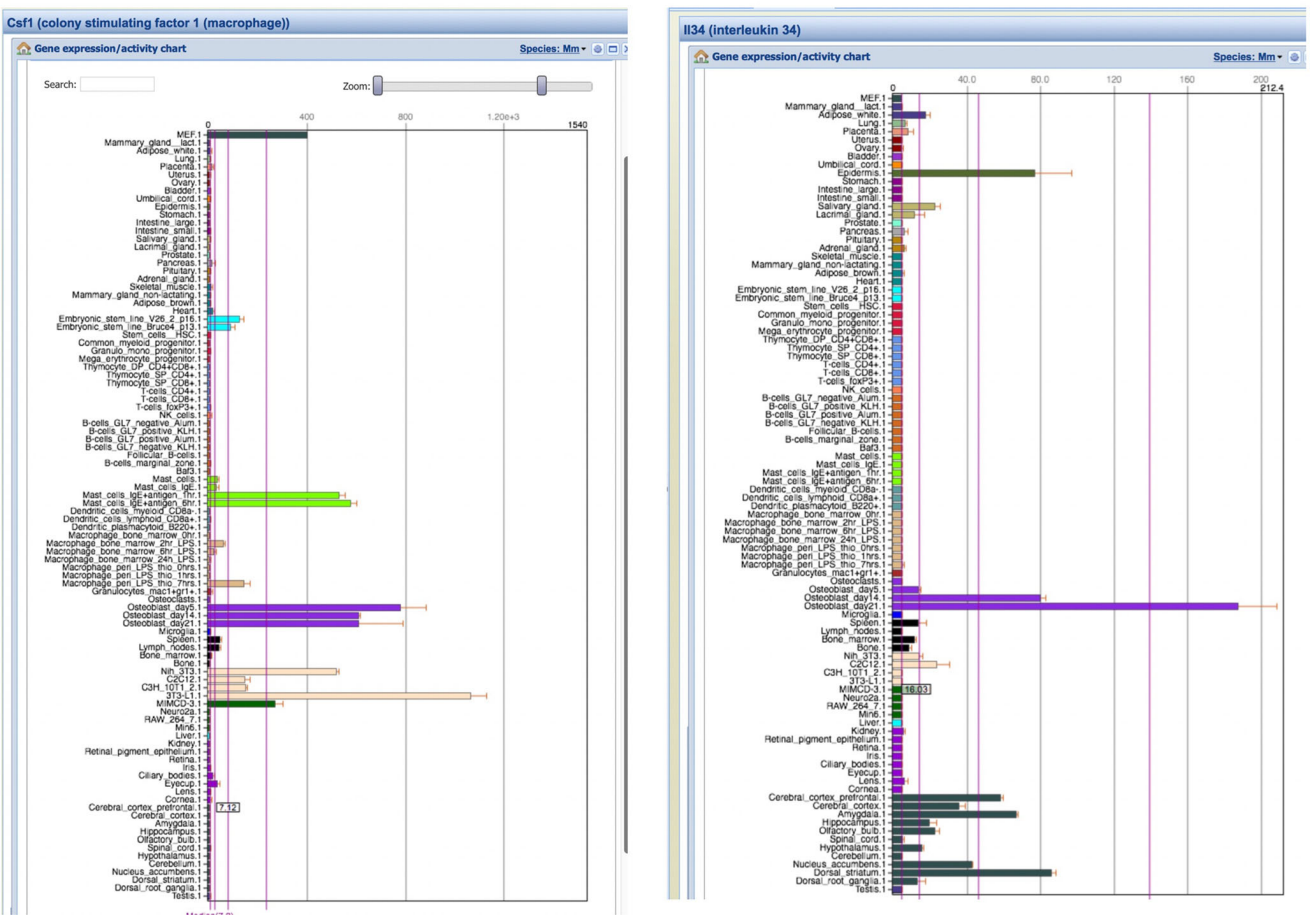
Expression of *Csf1* and *Il34* mRNA in mouse. Figure shows screenshots from BioGPS.org of the expression of *Csf1* and *Il34* mRNA in a wide range of mouse tissues and cells. *Csf1* is expressed in embryonic fibroblasts (MEF), ES cells, stimulated mast cells, osteoblasts, and various mesenchymal cell lines. As expected, Il34 was detected primarily in the epidermis and brain, but was also induced during differentiation of primary osteoblasts

*Csf1* mRNA is expressed widely in all species studied, predominantly in cells of mesenchymal lineages. *Il34* mRNA in humans is most highly expressed in the brain, spleen, and epidermis (see BioGPS.org; Gtex.org), with transcription initiated from distinct promoters [63]. Figure 2 shows expression profiles of the two regulators in mouse from BioGPS. These profiles highlighted the expression of *Csf1* by mast cells and the region-specific expression of *Il34* in the brain. More importantly, these data highlighted the induction of *Il34* mRNA during induced maturation and calcification in calvarial osteoblasts [44]. Expression of *Il34* by differentiating mouse calvarial osteoblasts was confirmed by others [64]. The promoter-based analysis by the FANTOM5 consortium [63] showed that many distinct types of mesenchymal cells express *Il34* from a third transcription start site. Few of these resources provide quantification and location of expression in bone in situ*.*

Ryan et al. [65] described the detection of a *Csf1* promoter-lacZ transgene in osteoblasts and fibroblasts specifically enriched on trabecular surfaces in bone in proximity to OCL. The major secreted and circulating form of CSF1 is a chondroitin sulphate proteoglycan, a biology that is conserved in birds [66]. Nandi et al. [67] reported that transgenic expression of the proteoglycan isoform, rather than cell surface or glycoprotein isoforms, was required to reverse the OCL deficiency and osteopetrosis in *op/op* mice [67, 68]. In rat bone marrow, the 4.6kb *Csf1* mRNA encoding the secreted CSF1 protein is abundant and readily detected by Northern blot [69]. Interestingly, the 1.4kb transcript encoding membrane CSF1 was induced following ovariectomy.

Conditional deletion of floxed *Csf1* alleles in C57BL/6 mice as well as the selective restoration of CSF1 expression in *op/op* mice supported the idea that local CSF1 production is essential for normal bone development (reviewed in [70]). There is some evidence for an effect of mouse genetic background on the penetrance of CSF1 mutations, and it is notable that C57BL/6J female develop early-onset osteoporosis [71]. Harris et al. [72] reported a generic deletion of *Csf1* in mesenchymal cells using *Meox*-cre was sufficient to drive marked reduction in OCL and tissue macrophages leading to osteopetrosis and impaired osteocyte differentiation and survival. Subsequent restricted deletion in osteocytes, using *Dmp1*-cre, produced little or no bone phenotype [73, 74] suggesting these cells are not a major source of growth factor. The existence of separate niches within marrow is suggested by deletion of *Csf1* expression in osteolineage and vascular compartments. Whereas conditional deletion in osteolineage cells using *Osx*-cre reproduced bone developmental abnormalities, deletion in the vascular compartment with *Cdh5*-cre had a selective effect on marrow monocytes [74]. Selective expression of *Csf1* and *Il34* is evident in mouse bone single-cell RNA-seq data. In profiles of bone cells cultured from developing calvaria [75•], *Csf1* mRNA was detected in all of the mesenchymal populations, whereas *Il34* was more restricted to chondrocytes and immature osteoblasts. As reported previously, *Csf1r*^*+*^*/Adgre1*^*+*^ macrophages were abundant in these cultures and lacked expression of either ligand. Low level cross contamination of some osteoblasts with *Csf1r* mRNA in the scRNA-seq data likely reflects adhesion of macrophage remnants [52]. The scRNA-seq data support adipocyte-primed leptin receptor positive (LEPR+) mesenchymal cells and sinusoidal endothelial cells [42, 43, 74] as sources of both *Csf1* and *Il34* in mouse marrow. In overview, all the available data suggests that local CSF1/IL34 is important, and likely mediates interactions between osteoblasts and CSF1R-expressing macrophages, OCL and progenitors as summarized schematically in Fig. 1.

## CSF1R Signals in Bone Development

The local production of CSF1 and the development of OCL each appear relatively late in mouse gestation [76]. In 17-day-old embryos, *Csf1* transcripts were present in cells lining the outside of the midregion of the metatarsals. At 18 days, *Csf1* transcripts were detected by in situ hybridization in newly mineralized cartilage. OCL precursors fuse and the mature OCL invades the mineralized cartilage of the rudiments to excavate the future bone marrow cavity. In these studies, CSF1 was considered a possible chemoattractant as well as a growth factor for OCL precursors. Given the late appearance in gestation, it is not surprising that skeletal development in *Csf1*- and *Csf1r*-deficient mice and rats appears relatively normal at birth; the major effects of the mutations appear in the postnatal period. In the case of CSF1 deficiency, transplacental transfer of the growth factor probably compensates for the loss in the embryo. Because of the relative infertility of the *op/op* and *tl/tl* females, homozygous mutant pups are derived from heterozygous matings and they are not entirely macrophage-deficient at birth [77]. However, CSF1R-deficient rat embryos are macrophage-deficient [78•] and in mouse the embryonic macrophage population can be depleted by anti-CSF1R administration to the mother [79]. So, professional phagocytes in the embryo are genuinely redundant. The phagocytic activities of the abundant macrophage population in the embryo are not restricted to bone; they are involved in apoptotic cell removal throughout the body [80]. Wood et al. [81] examined the basis for macrophage redundancy in the clearance of apoptotic cells from the interdigital spaces in the footpad of macrophage-deficient PU.1 knockout mice. In the absence of macrophages, phagocytosis was taken over by mesenchymal neighbors. These amateurs appeared somewhat less efficient at recognition engulfment and digestion of apoptotic debris than professionals, but the task was nevertheless completed in a relatively normal time frame and there was no accumulation of pyknotic nuclei.

Even the postnatal development of the skeleton is not completely compromised in animals that are macrophage- and OCL-deficient. Albeit with a much more extensive trabecular network and increased bone density, there is a bone marrow cavity with active hematopoiesis. Despite the absence of hematopoietic island macrophages in the fetal liver and bone marrow, which function in regulation of erythropoiesis and myelopoiesis (see Fig. 1) [82, 83], the mutant animals are not anemic and there is no accumulation of expelled red cell nuclei in the fetal liver. So, to some extent, amateurs must also fulfill some of the tasks normally undertaken by macrophages and OCL in bone development and hematopoiesis. In fact, it is not even certain that any of the impacts of the lack of macrophages and OCL reflect non-redundant functions within bone as opposed to indirect systemic effects. CSF1R signals intersect the regulation of somatic growth via the growth hormone-IGF1 axis and mutant animals are deficient in circulating IGF1 [78•, 84]

As mentioned above, there has been only one isolated report of recessive *CSF1* mutation in human osteopetrosis. However, subtle variants in CSF1 protein sequence could potentially alter binding to CSF1R. Such variants determine the species specificity of CSF1:CSF1R interaction. Mouse CSF1 binds poorly to human CSF1R whereas human CSF1 is active in mouse. Pig CSF1 is equally active in mouse and human [85–87]. A comparative analysis of the conservation of CSF1:CSF1R contact residues based on the crystal structure of the complex identified multiple candidate substitutions that might contribute to the species specificity [87]. Large-scale exome and genomic sequencing of human genomes (https://gnomad.broadinstitute.org) has identified numerous point mutations within the 150 amino acid bioactive human CSF1 core. They include receptor contact residues and amino acids that are conserved across all mammalian species. Without testing the biological activity of these allelic variants, we cannot determine whether there is any loss of function. However, given the expression of IL34 in bone, it is also possible that loss-of-function mutations in CSF1 in humans do not actually manifest in an overt bone phenotype. By contrast, bi-allelic *CSF1R* mutations in human patients are associated with skeletal dysplasia and osteosclerosis [88•, 89•]. The syndrome has been called “brain abnormalities, neurodegeneration, and dysosteosclerosis” (BANDDOS, OMIM # 618476; reviewed in [90]). Individuals surviving infancy probably have at least one hypomorphic allele and may not be entirely CSF1R-deficient [38]. No evidence of severe growth retardation seen in rodents has been reported in BANDDOS patients and at least some of the individuals had normal circulating TRAP5b, indicating the functional presence of OCL [88•].

The analysis of the bone phenotype in *Csf1r* mutant mice is compromised by the strain-dependent pre-weaning lethality. Both ligand and receptor mutations were associated with expanded epiphyseal chondrocyte region, severely disturbed matrix structure, and disorganized collagen fibrils [41, 91]. The layered organization of osteoblasts on the bone-forming surface and the direction of their matrix deposition appeared disrupted, and there was a defect in mineralization. Although these phenotypes were attributed to the lack of OCL, they are also consistent with the regulation of osteoblast differentiation by osteomacs [44].

**Fig. 3:**
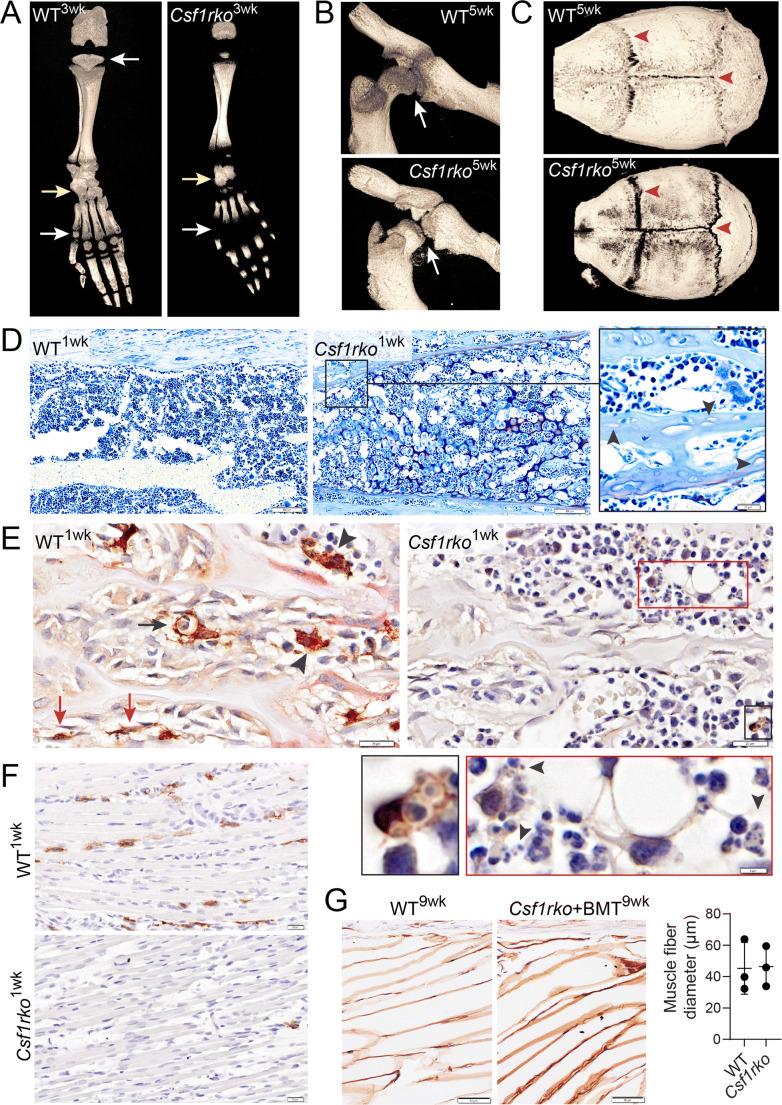
Phenotypic analysis of skeletal development in *Csf1r* knockout rats. Homozygous knockout mutation of *Csf1r* in rats (*Csf1rko*) was generated by Pridans et al. [11•] and impacts on skeletal development were described in [78•]. This figure contains previously unpublished images highlighting aspects of the mutant phenotype and its reversal by bone marrow cell transfer. **A**–**C** show μCT images demonstrating the delay in ossification in the digits and formation of the hip-joint and defective cranial suture closure. **D** shows the tibial diaphyseal region in juvenile WT and *Csf1rko* rats and the presence of empty osteocyte lacunae. The *Csf1rko* was associated with substantive loss of both OCL pink stained for expression of tartrate-resistant acid phosphatase (TRAP) and osteomacs (brown stained for expression of IBA1 (**E**). The inset shows that the residual macrophages showed evidence of efferocytosis but extracellular pyknotic nuclei are also evident (arrowheads). **F** shows that IBA1^+^ macrophages are also depleted in skeletal muscle in the *Csf1rko* rat associated with the reduction in muscle fiber diameter and postnatal somatic growth retardation that is observed in these animals [79]. Transfer of WT bone marrow (BMT) at weaning without conditioning corrected all of these musculoskeletal phenotypes, as exemplified by restoration of muscle mass and fiber diameter (**G**)

The effect of null *Csf1r* mutation (*Csf1rko*) has been analyzed in more detail in the rat [78•]. As shown in Fig. 3A, B**,** there is delay in subarticular ossification of short bones, secondary ossification center formation of long bones, and hip-joint formation. One phenotype that was not reported in mutant mice is a profound lack of mineralization in the cranial case and defective cranial suture closure (Fig. 3C) whereas the skull base is hyper-mineralized as it is in human bi-allelic mutations (leading in human to impacts on the cerebellum and the Dandy-Walker malformation). Flat bones are formed by intramembranous ossification, a process that unlike endochondral ossification involves condensation of mesenchymal stem cells (MSC) and their direct differentiation into osteoblasts [92]. Figure 3D highlights disorganization of the tibial diaphyseal region in juvenile *Csf1rko* rats. There is some evidence of conversion of the abnormal bone into “mature” cortical template but the more mature bone often contains empty osteocyte lacunae. The impact of the *Csf1rko* was associated with substantive loss of both OCL and osteomacs (Fig. 3E). Residual resident macrophages showed evidence of efferocytosis, but the marrow also showed evidence of accumulation of pyknotic nuclei.

IL34 is also expressed in bone, but thus far no biological function has been identified. No loss-of-function human IL34 mutation has been reported nor is there any evidence of association with IL34 alleles with bone phenotypes. Similarly, no skeletal phenotype or impact on somatic growth has been reported in the mouse IL34 knockout [93]. IL34 is more highly conserved across mammalian species than CSF1 [88•] and the gnomAD database (gnomad.broadinstitute.org) records numerous coding variants that impact highly conserved amino acids. Since, IL34 knockout mice are viable and fertile, it is possible that there are also bi-allelic mutations in humans with little overt phenotype. Baghdadi et al. [94] presented evidence that IL34 production by multiple myeloma cells contributes to tumor-associated osteolysis. In the rat, homozygous *Csf1r* mutation has a much more profound effect on somatic growth than *Csf1* mutation, implying a role for *Il34* [13], but both *Csf1* and *Csf1r* mutants are entirely OCL-deficient. Unlike *op/op* mice, *Csf1*^tl/tl^ rats show no evidence of age-dependent recovery of OCL [3], and indeed, there is no evidence of extramedullary hematopoiesis in the spleen.

## Rescue of the Effect of CSF1R Mutation by Bone Marrow Cell Transfer

Consistent with the lack of irreversible consequences of the lack of embryonic macrophages, the severe phenotypes observed in *Csf1r* knockout mice and rats can be rescued by postnatal intraperitoneal transfer of wild-type bone marrow cells without any conditioning [78•, 95]. In the rats, this rescue can be achieved as late as weaning, providing a unique model to dissect the precise roles of CSF1R-dependent cells in skeletal morphogenesis [78•]. Surprisingly, rescue is achieved without restoring the blood monocyte population or CSF1-responsive progenitor cells in the bone marrow. Donor bone marrow cells proliferate and differentiate to form macrophages in the peritoneal cavity and appear to traffic directly to distal sites throughout the body, including the brain. Donor-derived macrophages appeared prior to OCL in all of the affected locations in bone indicating that they provide the pioneering cells in primary and secondary ossification center formation, in cranial ossification and suture closure (Batoon et al. Manuscript submitted). In the bone marrow, both hematopoietic island macrophages and OCL were restored from cells of donor origin, whereas the hematopoietic compartment was populated by recipient progenitor cells. These findings in mice and rats support the view that OCL can be generated entirely from bone marrow without input from embryonic erythro-myeloid progenitors. Phenotypic rescue was associated with restoration of somatic growth and circulating IGF1 levels [78•]. CSF1R-dependent macrophages are also abundant in skeletal muscle, cartilage, and tendon and their loss in the *Csf1rko* was associated with reduction in muscle fiber diameter in juveniles (Fig. 3F and [79]). Wild-type BM transfer also restored muscle macrophage populations and muscle fiber diameter (Fig. 3G). The precise interplay between systemic and local impacts of the BM transfer in bone requires further investigation.

## CSF1R and Homeostasis

Tamoxifen-inducible Cre recombinase (ER2-cre) transgenic lines have been widely used in lineage trace models of macrophage and OCL ontogeny. One of the assumptions in studies of macrophage ontogeny is that tamoxifen is a neutral agonist that does not impact macrophage differentiation [96]. The toxicity of tamoxifen in OCL was reported many years ago [97]. In the process of developing a conditional deletion approach for OCL using OCL-specific promoters [98], we tested the effect of tamoxifen treatment at doses routinely used in inducible recombination on OCL in vivo. We found that initial ablation of OCL was followed by a rebound osteoclastogenesis and loss of trabecular bone (unpublished). The acute depletion of OCL by tamoxifen was confirmed recently, alongside a complex dose-dependence wherein low doses actually had the reverse effect [99•]. Current front-line treatments for osteoporosis, bisphosphonates [100] and antibodies against RANKL [101•], also deplete OCL. Cessation of treatment, especially with anti-RANKL, is associated with a rebound osteoporosis [102•].

CSF1 likely contributes to the rebound phenomenon. CSF1 (or IL34) binding to CSF1R is followed by receptor-mediated endocytosis and degradation of both ligand and receptor [103]. New receptors are constantly synthesized and trafficked to the cell surface so that in the steady state macrophages continuously internalize and degrade their ligand. Receptor-mediated endocytosis by macrophages in the liver and spleen controls the circulating CSF1 concentration [104]. As a consequence, CSF1 is elevated in the circulation in CSF1R-deficient animals and in response to anti-CSF1R antibody treatment [10, 78•, 105, 106]. When ligand is removed, CSF1R accumulates on the cell surface and the expression of CSF1R target genes declines rapidly [107, 108]. Interestingly, CSF1R is removed from the cell surface by ectodomain cleavage in response to various toll-like receptor agonists [109]. This could provide a mechanism for acute local or systemic increases in CSF1 in the absence of increased synthesis.

We and others have proposed that macrophages also control the local availability of their own growth factor within tissues as well as systemically [96, 110]. Consequently, when CSF1R-positive cells are depleted locally, there is an intrinsic drive to restore the homeostatic distribution. By the same token, an excess of CSF1R-positive cells in any tissue is unsustainable unless local or systemic CSF1 availability is increased. One clear example of increased ligand availability is the effect of female hormones on *Csf1* transcription [69, 111]. In the specific case of bone, the question is, how local is local? There are multiple CSF1R-positive cells in marrow: monocytes and their progenitors, osteomacs, hematopoietic island macrophages, and OCL (Fig. 1; [112]). These populations could potentially compete with each other for available growth factor.

## CSF1R-Directed Therapy: More or Less or Both?

The relationship between systemic and local CSF1, the potential role of IL34, the diversity of target cells in bone, species specificity, and the intrinsic homeostatic mechanisms each need to be considered in the development of optimal treatment regimes for CSF1R-direct therapies. Administration of recombinant CSF1 was sufficient to reverse/correct many of the bone developmental phenotypes in *op/op* mice and *tl/tl* rats as well as liver and splenic macrophage populations [77, 113–115]. The treatment was less effective at restoring resident macrophage populations in other locations, somatic growth, and female fertility [77, 115]. The dose-dependence of rescue by exogenous CSF1 was not analyzed. By adulthood, the dose applied may not have been sufficient to saturate clearance by the liver and spleen. CSF1 administered systemically is probably also selectively accessible to the bone marrow, which shares with the liver a sinusoidal endothelial lining permitting blood cell egress [116].

The first report of CSF1 administration to mice documented an expansion of the CSF1-responsive marrow cell population as well as a monocytosis [85]. The potential of recombinant CSF1 as a therapeutic agent was constrained by the short half-life; the bioactive dimeric recombinant protein is well below the clearance threshold so that in early clinical trials continuous infusion was required. This issue was addressed by the generation and characterization of a CSF1-Fc fusion protein [117] which has been tested in a range of preclinical studies. Daily treatment for 4 days produced a monocytosis and increased liver and spleen mass that peaked around 7–10 days and was rapidly reversed [86, 117, 118]. CSF1-Fc treatment also caused an expansion of mature OCL populations on trabecular bone surfaces and the epiphyseal plate. In the short time frame of these studies, there was no change in bone density or trabecular architecture. However, Lloyd et al. [119, 120] found that prolonged high-dose daily administration of CSF1 to mice increased serum bone turnover markers but nevertheless produced an anabolic effect attributed to the coupled activation of osteoblasts. The same phenomenon was observed in chickens treated with an equivalent avian CSF1-Fc protein [21]. The interpretation of CSF1 response based upon OCL-osteoblast coupling neglects the expansion and stimulation of osteomacs, which likely act directly to promote osteoblast function [44]. The anabolic effects of CSF1 have been harnessed to promote intramembranous ossification in models of bone repair [45•, 121•]. CSF1-Fc dosing can be titrated to increase osteomacs without impacting osteoclast number [121•]. While CSF1-Fc did not reverse the age-dependent osteopenic phenotype observed in female C57BL/6J mice, it promoted a pro-anabolic response post-fracture in both healthy and osteoporotic bones [121•]. The effect was influenced by age and gender [121•], reinforcing the importance of considering these variables when investigating CSF1/CSF1R-directed therapies.

There have been few reports of the effect of IL34 administration. IL34 can replace CSF1 for the generation of OCL from mouse bone marrow progenitors or human monocytes in vitro [64]. Daily IP injections of a relatively low dose of recombinant IL34 increased myeloid cell populations in the marrow and reduce trabecular volume, but the effect on OCL numbers was not reported. IL34 has an affinity for other potential receptors (PTPRZ, CD138) through binding to proteoglycans [122] which may influence the in vivo pharmacology.

Several studies have targeted CSF1/CSF1R signaling to reduce OCL number or function to mitigate bone loss. CSF1R kinase inhibitors and anti-CSF1R antibodies have been evaluated for therapeutic intervention in diseases where macrophages contribute to pathology, e.g., neurodegenerative disease, cancer, inflammation, and fibrosis [123•, 124]. Relatively few studies have focused on bone. PLX3397, which inhibits CSF1R and related kinases, was reported to reduce LPS-induced osteolysis, but the effects were small and OCL were largely retained [125, 126]. Similarly, He et al. [127] found that PLX3397 inhibited CSF1-dependent excess osteoclastogenesis in a mouse neurofibromatosis model in response to ovariectomy. The dose was high (80 mg/kg) but the effects were small and OCL were only marginally altered. Prolonged treatment with another widely applied CSF1R kinase inhibitor, GW2580, inhibited OCL function in vitro and was effective in various inflammatory models [128, 129] but OCL depletion was not observed in vivo. Gleevec (Imatinib), which is in clinical use for treatment of chronic myelogenous leukemia, is also an inhibitor of CSF1R kinase activity, albeit less specific, and effects on OCL may contribute to dysregulated calcium/phosphate homeostasis in patients [130, 131]. In overview, OCL and peripheral macrophage populations are relatively resistant to CSF1R kinase inhibitors. This is partly because macrophage/OCL survival requires lower threshold signal than proliferation, so a drug must provide almost complete and sustained inhibition of CSF1R kinase activity to be effective. It is also likely a consequence of intrinsic homeostasis, where any loss of CSF1R-positive cells drives the local CSF1 concentration higher and promotes compensatory proliferation.

Blocking antibodies are potentially more effective because they can saturate surface receptors and have more favorable half-life/pharmacokinetics. Both anti-CSF1R and anti-CSF1 antibodies have been found to ablate OCL and/or inhibit OCL activity with consequent reduction in bone loss in mice [50, 71, 132–134]. The effects seem to be specific to excess OCL activity. For example, whereas prolonged anti-CSF1R depletion of OCL prevented spontaneous bone loss in female C57BL/6J mice, it did not alter bone density in males [71]. Neonatal treatment of mice with anti-CSF1 was sufficient to phenocopy effects of the op/op mutation on bone [135]. Cenci et al. [111] described increased OCL numbers in an *Egr1* mutant mouse line, attributed to increased local production of CSF1. A neutralizing antibody to CSF1 restored rates of bone resorption to normal in mutant animals and also completely prevented ovariectomy-induced bone loss in control animals. Anti-CSF1 or anti-CSF1R antibodies have also been evaluated in patients with the focus on inflammatory disease and on depleting tumor-associated macrophages [123•]. Administration of the humanized anti-CSF1R antibody AMG820 in cancer patients produced sustained elevation of circulating CSF1 and ablation of dermal macrophages. There was no effect on circulating TRAP5b, considered a marker of OCL activity [106, 136]. Similarly, a detailed Phase 1 trial of a neutralizing anti-CSF1 antibody in a large cohort of volunteers demonstrated no effect on TRAP5b in peripheral blood, despite a clear dose-dependent decrease in circulating blood monocyte populations [137•]. However, they did observe a reduction in detectable CTX-1, an alternative marker of active resorption. The recipients of anti-CSF1 treatment exhibited periorbital swelling in the eye. In parallel non-human primate studies, this was associated with accumulation of basophilic materials (mainly hyaluronic acid glycosaminoglycans). They reported similar accumulation in multiple other organs. Periorbital swelling is also observed in patients treated with anti-CSF1R [123•] but the mechanism remains unknown. The relative lack of effect of anti-CSF1 contrasts with the ability of anti-RANKL (Denosumab) to produce a 40–50% decline in TRAP5b at least in individuals with elevated TRAP5b associated with osteoporosis [138, 139]. Prolonged anti-RANKL treatment did produce a decline in OCL-specific transcripts in subsequent bone marrow biopsies [140]. One consequence of prolonged OCL depletion was a decrease in circulating dipeptidyl peptidase 4 and an increase in glucagon-like peptide 1, which the authors speculate provides a link between bone and energy metabolism.

There are caveats in comparing effects of anti-CSF1R in animal models and humans. In the presence of anti-CSF1R, CSF1 rises locally and systemically to produce a new steady state where ligand may compete for binding to the receptor. The relative affinity of antibody and ligand for the receptor determines this set point and in turn whether macrophage and OCL populations can be depleted. There is some evidence for differences between rodents and humans. In humans, the circulating CSF1 concentration was very low (<50pg/ml); with a maximal dose of AMG820, it increased 10^5^-fold, to 1000ng/ml [106]. Circulating CSF1 appears higher (around 5ng/ml) in rodents and increased only 20–50-fold in anti-CSF1R-treated mice [105] or in *Csf1rko* rats [78•]. These findings suggest the human CSF1R has a higher affinity for ligand, and consequently, it may be more difficult to achieve sustainable blockade with anti-CSF1R antibodies. A second important difference between mouse and human lies in the regulation of CSF1 expression. Whereas mouse macrophages are dependent on exogenous ligand, in all other species (including rats and humans) CSF1 is expressed constitutively by macrophages themselves [70]. Autocrine CSF1/CSF1R signaling in OCL in humans may be less accessible to inhibition by antibodies.

## Conclusions

Anti-CSF1R or anti-CSF1 treatment is reasonably well-tolerated, albeit with significant side effects including periorbital edema noted above [123, 137•]. There may be applications in acute bone loss associated with infection, bone metastasis, or glucocorticoid treatment. Paradoxically, CSF1 treatment may produce similar outcomes. In both cases, the effects are likely to be rapidly reversible because of the intrinsic homeostasis. Indeed, promising clinical trials of anti-CSF1R in chronic graft versus host disease have been based on a repeated intermittent treatment [123•]. In overview, while there is no doubt that CSF1/CSF1R signals control OCL and macrophage function in bone during development, the therapeutic applications of this knowledge remain unclear.
